# Author Correction: Targeted gadofullerene for sensitive magnetic resonance imaging and risk-stratification of breast cancer

**DOI:** 10.1038/s41467-017-02302-9

**Published:** 2018-01-08

**Authors:** Zheng Han, Xiaohui Wu, Sarah Roelle, Chuheng Chen, William P. Schiemann, Zheng-Rong Lu

**Affiliations:** 1 Case Center for Biomolecular Engineering, Department of Biomedical Engineering, Case Western Reserve University, 10900 Euclid Avenue, Cleveland, OH 44106 USA; 2 Case Comprehensive Cancer Center, Case Western Reserve University, Cleveland, OH 44106 USA

Correction to: *Nature Communications* 10.1038/s41467-017-00741-y, Article published online 25 September 2017

In the original version of this Article, the penultimate sentence of the Abstract incorrectly read ‘The dose of the contrast agent for effective molecular MRI is only slightly lower than that of ZD2-Cy5.5 (0.5 µmol kg^−1^) in fluorescence imaging.’ The correct version states ‘higher’ in place of ‘lower’. This error has been corrected in both the PDF and HTML versions of the Article.

