# Supplementary material for: Targeted gadofullerene for sensitive magnetic resonance imaging and risk-stratification of breast cancer
Source: Nat Commun. 2017 Sep 25;8:692. doi: 10.1038/s41467-017-00741-y (PMC5612990; doi:10.1038/s41467-017-00741-y)
Supplement: Supplementary file 1 — Supplementary Information [file 41467_2017_741_MOESM1_ESM.pdf]

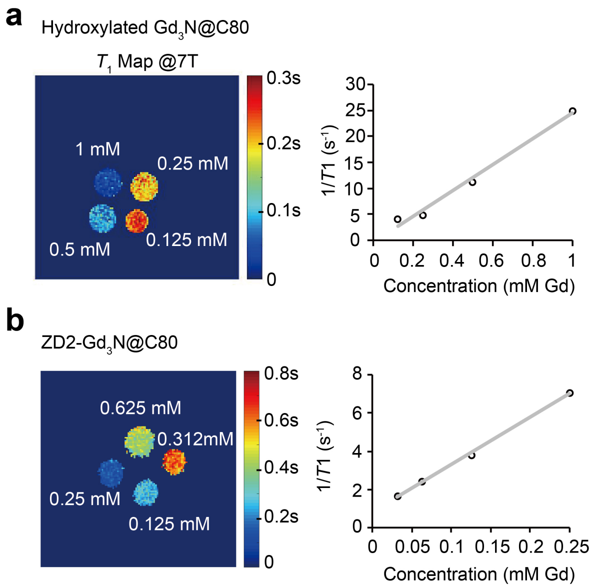

Supplementary Figure 1 | Relaxivity measurement of hydroxylated $Gd_3N@C80$ (**a**) and ZD2-$Gd_3N@C80$ (**b**) at 7 Tesla. T_1 maps of NMR tubes containing gradient concentrations of contrast agents, and plots of $1/T_1$ versus concentrations are shown. The r_1 relaxivities derived from the plots were r_1 = 24.68 $mM^{-1}s^{-1}$ per Gd for hydroxylated $Gd_3N@C80$ and r_1 = 24.78 $mM^{-1}s^{-1}$ per Gd(III) for ZD2-$Gd_3N@C80$.

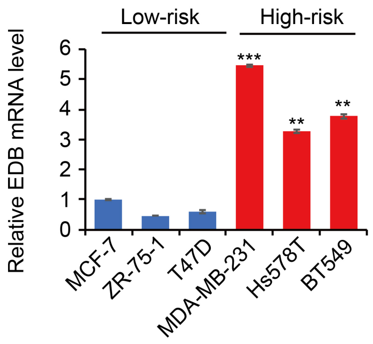

Supplementary Figure 2 | RT-PCR analysis of EDB-RNA levels in breast cancer cells. MDA-MB-231, Hs578t and BT549 cells are high-risk TNBCs. MCF-7, ZR-75-1 and T47D are ER-positive breast cancer cells. Data are represented as ratios to the EDB mRNA level of MCF-7. The mRNA level of β-actin was used for normalization. **: $P<0.01$ and ***: $P<0.001$ compared to low-risk breast cancer cell lines (two-tailed *t*-test; Data are presented as mean±s.e.m. n=3-4).

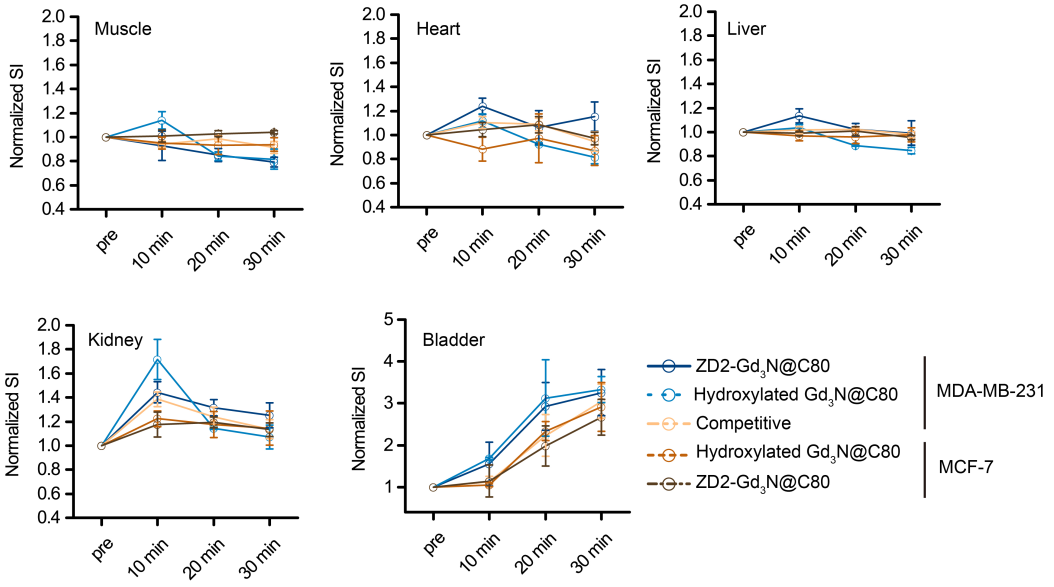

Supplementary Figure 3 | Dynamic change of normalized signal intensities in muscle, heart, liver, kidney, and bladder in MDA-MB-231 and MCF-7 tumor models after injection of 5 µmol Gd/kg ZD2-Gd_3N@C80, Gd_3N@C80, or the mixture of 25 µmol/kg free ZD2 and 5 µmol Gd/kg Gd_3N@C80 (competitive) (Data are presented as mean±s.e.m. n=3).

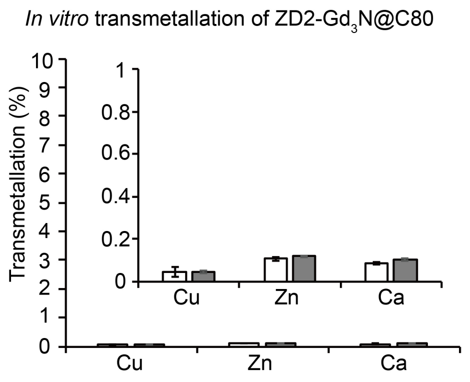

Supplementary Figure 4 | *In vitro* transmetallation assay of ZD2-Gd_3N@C80 after incubation in human serum for two hours. Data are presented as mean±s.e.m.

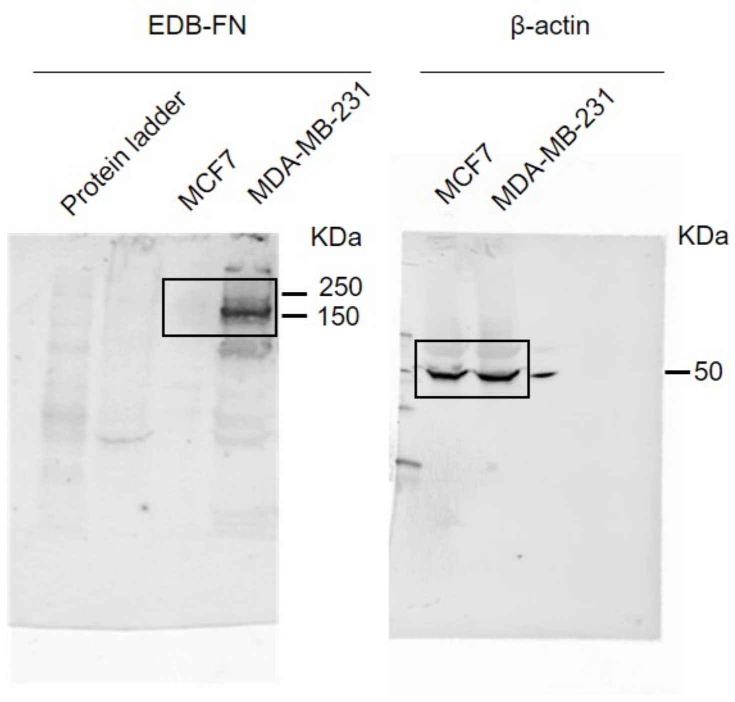

Supplementary Figure 5. Western blot with full gel and protein ladder showing EDB-FN and β-actin expression in MCF-7 and MDA-MB-231 tumours of Fig 3b.
